# Effects of 12 Months of Structured Physical Activity Program and 18-Month Follow-Up Period on Body Composition, Physical Capacities, and Physical Activity Levels in Adults with Obesity

**DOI:** 10.3390/ijerph22050665

**Published:** 2025-04-23

**Authors:** Lara Mari, Mattia D’Alleva, Francesco Graniero, Valeria Azzini, Federica Fiori, Michela Marinoni, Maria De Martino, Enrico Rejc, Simone Zaccaron, Jacopo Stafuzza, Miriam Isola, Maria Parpinel, Stefano Lazzer

**Affiliations:** 1 Department of Medicine, University of Udine, 33100 Udine, Italy; mari.lara@spes.uniud.it (L.M.); dalleva.mattia@spes.uniud.it (M.D.); federica.fiori@uniud.it (F.F.); michela.marinoni@uniud.it (M.M.); maria.demartino@uniud.it (M.D.M.); enrico.rejc@uniud.it (E.R.); simone.zaccaron@uniud.it (S.Z.); jacopo.stafuzza@uniud.it (J.S.); miriam.isola@uniud.it (M.I.); maria.parpinel@uniud.it (M.P.); 2 School of Sport Sciences, University of Udine, 33100 Udine, Italy; 3 Physical Exercise Prescription Center, Azienda Sanitaria Universitaria Friuli Centrale, 33013 Udine, Italy; francesco.graniero@asufc.sanita.fvg.it (F.G.); valeria.azzini@asufc.sanita.fvg.it (V.A.); 4 Department of Neurosciences, Biomedicine and Movement Sciences, University of Verona, 37124 Verona, Italy

**Keywords:** body composition, maximal oxygen uptake, physical activity, obesity

## Abstract

(1) Background: Individuals with obesity tend to stop exercising after the completion of a structured training program. Thus, the aim of the present study was to assess adherence and body composition, cardiorespiratory fitness, physical activity levels, and physical and mental health after a 12-month exercise program and an 18-month follow-up period in a group of male adults with obesity. (2) Methods: Thirty-four adults with obesity were evaluated before (T0) and after (T3) a 3-month combined training (COMB). After that, they followed a maintenance program with low-intensity aerobic activity for three months. Then, they were recalled for a 6-month training program including thresholds (THR) training until the end of the study (T12). Finally, they participated in a 18-months follow-up period that included suggestions for healthy lifestyles, which ended with assessments (T30). Thus, the assessments were carried out at the beginning and end of the first training protocol (T0 and T3), at the beginning and end of the second training protocol (T6 and T12) and 18 months after the end of the training program (T30). At all time points, body composition (i.e., BMI, fat mass [FM] and fat-free mass [FFM]), physical capacities (i.e., V̇O_2_max), and physical habits (i.e., International Physical Activity Questionnaire (IPAQ) and Short-Form 12 (SF-12, for physical, P, and mental, M, indices) were measured. (3) Results: Fifteen out of thirty-four participants (42.8%) (age 42.8 ± 8.1 y) completed this study. At T30, participants increased their V̇O_2_max (3.07 ± 0.46 vs. 3.67 ± 0.60 L·min^−1^, *p* < 0.001), IPAQ TOT score (396 (2888) vs. 1356 (9144), *p* = 0.006), and SF12_MI score (41.1 ± 8.9 pt vs. 48.6 ± 11.0 pt) compared to T0. Furthermore, multivariate analysis showed that decrease in BMI was largely associated with the increase in the SF 12_PI questionnaire (0.032). Similarly, the decrease in %FM and the increase in V̇O_2_max were related with the increase in IPAQ tot (*p* < 0.001) and SF 12_MI (*p* < 0.001) scores. (4) Conclusions: 42.8% (15 out of 34) of the initial participants completed the follow-up test at T30 and maintained higher V̇O_2_max values than at T0. Conversely, their physical characteristics returned to baseline. The improvement in V̇O_2_max, compared to T0, correlated with maintaining high activity levels and with improved physical and mental well-being. In summary, it is recommended that people with obesity follow a structured physical activity program, as this leads to an improvement in physical capacities and physical and mental well-being. A personalized and monitored approach can lead to greater adherence to treatment and more effective long-term outcomes.

## 1. Introduction

In the last ten years, the prevalence of obesity increased exponentially, to the point where 38% of the global population is overweight or obese [1]. Overweight and obesity are associated with a variety of cardio-metabolic complications such as type II diabetes, hypertension and ischaemic heart disease, which in turn are associated with increased mortality for cardiovascular and all-cause mortality [2]. Adults with obesity and higher cardiorespiratory fitness (CRF) levels have a lower risk of morbidity and mortality than inactive obese and lean individuals [3], and a similar rate of fat oxidation compared to lean individuals with the same CRF [3,4]. Thus, to avoid serious health consequences, people with obesity need to reduce or eliminate risk factors related to diet, sedentary habits, and physical activity levels [5].

Aerobic training has been shown to be more effective than strength training in improving body composition and aerobic physical capacities in people with obesity [6]. Moderate intensity continuous training (MICT) is the gold standard method for improving the cardiometabolic profile of overweight adults and adults with obesity [7]. However, in the last 10 years, high-intensity interval training (HIIT) has been proposed as an alternative to MICT to improve body composition and physical capacities in adults with obesity [7]. HIIT consists of alternating short periods of high-intensity [i.e., lasting 1–4 min per step at an intensity ≥85% hear rate max (HRmax) and ≥80% of maximal oxygen uptake (V̇O_2_max)] with periods of low-intensity activity [4]. Both types of exercise (e.g., HIIT and MICT) have been shown to be equally effective in improving body composition in adults with obesity [8,9]. On the contrary, some studies have shown that HIIT may be better than MICT for improving CRF in this population [7,10,11].

The American College of Sports Medicine (ACSM) physical activity guidelines suggest that a combination of higher and moderate aerobic activity is an effective strategy for improving body composition and physical capacities in people with obesity, compared to HIIT and MICT alone [12]. Indeed, recent studies have shown that combining HIIT and MICT (COMB) with a polarized approach (POL: 70–80% of the total weekly training volume below the gas exchange threshold (GET), 5–10% of the work between the GET and the respiratory compensation point (RCP) and the remaining 10–15% above the RCP) [13] improved body composition, physical capacities, and fat oxidation rate similarly to HIIT and MICT alone [14,15]. In addition, COMB training was perceived as less intense than HIIT, which was reflected in the lower values of heart rate (HR) observed during the weekly training program [14,15]. It appears that a high volume of exercise below gas exchange thresholds (GET) and a lower volume of work above the respiratory compensation point (RCP) may be a better strategy to enhance aerobic metabolism (i.e., higher mitochondrial content and better mitochondrial function) in athletic and non-athletic populations [16,17,18,19].

Several studies have shown that physical activity was discontinued after the end of a structured training program [20]. Vaccari et al. [11] observed that after a 4-month follow-up period followed by a 3-month HIIT or MICT training intervention, only 72% of participants returned to the follow-up tests. Body mass (BM) and fat mass (FM) increased compared to the end of the training program, but were still lower (e.g., −4% and −20%) compared to baseline values in both groups. At the same time, the V̇O_2_peak remained higher (e.g., +8) in both groups at follow-up compared to the baseline values. During the follow-up period, the participants increased the total level of physical activity, which at least partially explains the maintenance of CRF levels [11]. Quist et al. [21] showed that 6 months after the end of a structured physical activity program, 62% of the participants returned for the follow-up examination [21]. Participants in the vigorous training group-maintained improvements in body composition and V̇O_2_peak compared to the moderate training group [21]. On the contrary, Rosenkilde et al. [22] showed that 77% of participants in follow-up tests did not maintain the positive effects induced by the intervention periods 1 year after the end of the 3-month intervention. The meta-analysis of Anderson et al. [23] showed that 4 or 5 years after the end of a structured weight loss program, maintenance of weight loss was 3 kg or 23% of initial weight loss and the rate of adherence to follow-up at 4 and 5 years was 55.4% and 79.7%, respectively [24]. Christiansen et al. [25], showed that a weight recovery of 13.2 ± 19 kg was observed after a follow-up period of 2 and 4 years followed by an intensive weight loss program. Only 5.3% of these participants maintained their weight loss after the program, with no difference between men and women [25].

Analysis of data from previous studies evaluating follow-up after endurance training revealed that (i) MICT was perceived as boring during the exercise intervention, whereas HIIT was perceived as more fun and time-saving [26], (ii) HIIT was perceived to increase the occurrence of unpleasant events during and after exercise, such as chest pain or asthma [27], (iii) follow-up was made in a short period of time (i.e., less than one year) or after a long period (i.e., five to six years), and (iv) the dropout rate at follow-up varies considerably between studies. Thus, to our knowledge, there is no study that examined an intermediate follow-up period (i.e., between one and three years) after an exercise intervention considering the combination of HIIT and MICT during the training period.

Therefore, the aim of the present study was to assess body composition, cardiorespiratory fitness, physical activity levels, and physical and mental health 18 months after the end of a 12-month aerobic exercise training program (COMB) in a group of male adults with obesity. After 18 months of follow-up, we expect that participants who remained active kept their V̇O_2_max and questionnaire scores high.

## 2. Material and Methods

### 2.1. Participants

Thirty-four male adults with obesity (BM: 105.3 ± 13.2 kg, FM (%): 38.8 ± 4.1%, V̇O_2_max: 47.2 ± 5.8 mL·kg·FFM^−1^·min^−1^) were recruited to participate in the project. All volunteers had a complete medical history and underwent physical and nutritional examinations. None of the subjects had cardiovascular, respiratory, neurological, skeletal, or metabolic and/or endocrine diseases, and none of the subjects were on regular medication or taking medication known to affect energy metabolism. The study was approved by the Ethics Committee of the Friuli-Venezia-Giulia Region (Italy) (protocol number 1764). The participants were identified with an anonymous identification code and the data were kept by the two principal investigators. Before the study began, the purpose and objective were carefully explained to each participant, and written informed consent was obtained.

### 2.2. Experimental Design

**Figure 1:**
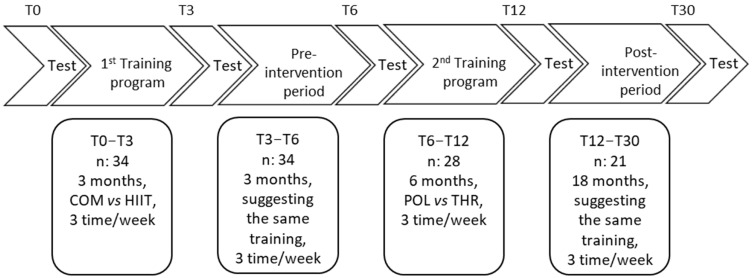
Schematic representation of the study design (T0: start; T3: 3rd month; T6: 6th month; T12: 12th month; T30: 30th month; and n: number of participants).

The first training intervention [15] was performed from May 2021 (T0) to September 2021 (T3) (Figure 1). Participants followed a 3-month weight management program involving two types of physical training programs (i.e., COMB vs. HIIT). Between the first and the second study (i.e., from September 2021 to November 2021) a 3-month pre-intervention period was employed to standardize the training load for all volunteers, suggesting the same amount of physical activity for all (i.e., from 100 to 200 min during week 1 performed at low-intensity running speed corresponding to 60% of V̇O_2_peak). The second training interventional [14] was performed from November 2021 (T6) to May 2022 (T12) (6 months). Participants followed a 6-month training program involving two types of physical training (i.e., thresholds (THR) vs. POL [14]). Testing sessions, performed before and after each training intervention, were conducted during one day and included assessment of anthropometric characteristics, body composition, V̇O_2_max, and physical and dietary habits. The participants were trained in their normal living conditions, ensuring the ecological validity of the study. During the 18-month follow-up period, the participants were invited to perform three training session per week: one at high intensity (90% HRpeak and less than 30 min), and two at low intensity (<70% HRpeak and more than 60 min) [11]. Eighteen months (T30) after the end of the second study [14], control tests, including assessment of body composition, physical capacities, physical activities, and dietary habits, were performed. In each phase of the project, participants were given a specific training program to follow and dietary recommendations to choose from.

The COMB group performed 86.0 ± 1.0% of the total training volume at low intensity (i.e., below the GET) and 14.0 ± 1.0% at high intensity (i.e., above the GET). The HIIT group spent 62.0 ± 2.0% of the total training at low intensity and 38.0 ± 2.0% at high intensity. The POL group spent 91.0 ± 2.4(%) of time at low intensity and the remaining amount of training above the GET. The THR group spent 71.3 ± 9.6% of weekly time at low intensity, and the remaining percentage of training above the GET (see [14] for further details).

### 2.3. Training Protocols

There were four different training protocols during the study: COMB, HIIT, THR, and POL. All exercise protocols included walking/running in a flat terrain and in an outdoor environment.

The COMB group performed 86.0 ± 1.0% of the total training volume at low intensity (i.e., below the GET) and 14.0 ± 1.0% at high intensity (i.e., above the GET).

The HIIT group spent 62.0 ± 2.0% of the total training at low intensity and 38.0 ± 2.0% at high intensity.

The POL group spent 91.0 ± 2.4(%) of time at low intensity and the remaining amount of training above the GET.

The THR group spent 71.3 ± 9.6% of weekly time at low intensity, and the remaining percentage of training above the GET (see [14] for further details).

### 2.4. Measurements

#### 2.4.1. Anthropometric Characteristics and Body Composition

The BM was measured with an electronic scale (approximate value 0.1 kg) (Seca 709, Hamburg, Germany), with the subject wearing only light underwear and no shoes. A height board mounted on the wall was used to measure stature. BMI was calculated as BM (kg) × stature^−2^ (m). Body composition was measured using bioelectrical impedance analysis (BIA, Human IM Plus; DS 171 Dietosystem, Milan, Italy) [28]. The values for fat mass (FM) and fat-free mass (FFM) were determined using the equations described by Grey et al. [29].

#### 2.4.2. Graded Exercise Test (GRAD)

We determined V̇O_2_max during a graded exercise test on a motorized treadmill (H/P/Cosmos Sports and Medical GmbH, Nussdorf-Traunstein, Germany). Each participant started the test at 5 km h^−1^ with a fixed slope at 1%. Then, the speed was increased by 0.5 km·h^−1^ every minute until the participant voluntarily exhausted [15]. All participants avoided strenuous exercise the day before the test and maintained the same eating habits. They came to the laboratory after a 12 h fast. During all tests, oxygen consumption (O_2_) and carbon dioxide production (V̇CO_2_) were continuously measured by indirect calorimetry (CPET, Cosmed, Rome, Italy). Before each test, we calibrated the gas analyzers and the flowmeter as recommended by the manufacturer. Throughout the test, an electrocardiogram was continuously recorded and displayed online for visual monitoring, and heart rate (HR) was recorded using a dedicated monitor (Garmin, Olathe, KS, USA). Perceived effort was assessed using the Borg scale [30]. V̇O_2_max was calculated as the average 30 s V̇O_2_ according to previously established criteria [31]: (i) plateau in V̇O_2_ (i.e., increase < 150 mL·min^−1^), (ii) respiratory exchange ratio (RER) > 1.1, and (iii) ≥90% of theoretical maximal heart rate.

#### 2.4.3. Dietary and Physical Activity Habits

Participants were asked to complete a 4-day dietary record (4-dDR) in which food and drink consumption was recorded on 2 weekdays and 2 weekend days at all the time points. The 4-dDR was used to provide instructions on how to record the type and portion size of food consumed in the first two studies. In this protocol, we asked the participants to send us their food diaries before they came to the hospital for completing tests. Intakes of selected macro and micronutrients were recorded after uploading the individual food information from the 4-dDRs into the Microdiet V4.4.1 software (Microdiet software-Downle Systems Ltd., High, Peak, UK), which contains the Italian “Food composition database for epidemiological studies in Italy” [32], together with information from nutritional labels, if required.

To assess the level of physical activity, we used the validated International Physical Activity Questionnaire Short Form (IPAQ-SF) [33]. The questionnaire measures total weekly physical intensity, moderate intensity, walking activity, and time spent sitting in the last 7 days. The IPAQ-SF scores were converted to metabolic equivalent minutes per week (MET-min·week^−1^) using the ‘Guidelines for Data Processing and Analysis of the International Physical Activity Questionnaire (IPAQ)’ [33]. In addition, all participants completed the Short-Form 12 (SF12) questionnaire to assess health-related quality of life. The questionnaire consists of 12 items from which physical (SF12_PI) and mental health indices (SF12_MI) are determined [34].

### 2.5. Statistical Analyses

Data were analyzed using GraphPad Prism version 10.0.1 (IBM, Chicago, IL, USA), with significance set at *p* < 0.05. Shapiro–Wilk was used to verify the normal distribution of the data. Sphericity was verified by Mauchly’s test. If the sphericity assumption was violated, the Greenhouse–Geisser correction was applied. All results are expressed as mean and standard deviation (SD) while in the case of a non-normal distribution, the data were expressed as median and interquartile range. Changes in body composition, V̇O_2_peak, and data derived from questionnaires and food diaries were assessed with a general linear mixed model that included the repeated measures analysis at the different time points. Analysis of the data of body composition, V̇O_2_peak, and data derived from questionnaires and food diaries only in the participants in the follow-up group and at the different time points were assessed with ANOVA for repeated measures. Post hoc comparisons were performed using the Bonferroni procedure for significant differences. The Friedman test was used to assess differences in non-normally distributed parameters between the different time points with post hoc analysis with the Nemenyi test. Pearson or Spearman correlations were used to analyze the possible relationships between baseline anthropometric characteristics, physical capacities, and data derived from questionnaires and food diaries. To evaluate the totality of participants for each time point, we did perform a mixed effect model with maximum likelihood estimation in case of missing values. A linear mixed model with a univariable and multivariable analysis was used to analyze the possible relationships between baseline anthropometric characteristics, physical capacities, and data derived from questionnaires and food diaries considering all the time points [35]. All variables with a *p*-value less than 0.10 in the univariate analysis were included in the multivariate model. Finally, effect sizes comparing pre–post changes within blood parameters were calculated as the corrected effect size (ES) [36,37,38]. ES < 0.20 was considered small, <0.50 medium, and >0.50 large 

## 3. Results

### 3.1. Characteristics of All Participants Assessed in This Study

Considering all participants enrolled at each time point, we showed that BM was significantly lower at T12 compared to T6 by 2.7 ± 3.24 kg (ES:0.24, small, *p* = 0.011), T3 by 3.7 ± 4.42 kg (ES:0.51, large, *p* = 0.024), and T0 by 6.8 ± 6.40 kg (ES:0.77, large, *p* = 0.003), but at T30, it returned similarly to baseline values (*p* > 0.05). BM was lower also at T3 compared to T0 by 3.1 ± 3.2 kg (ES:0.23, small, *p* < 0.001) and at T6 compared to T0 by 4.2 ± 4.2 kg (ES:0.56, large, *p* = 0.005).

FM (kg) decreased at T12 compared to T6 by 3.3 ± 3.1 kg (ES:0.49, medium, *p* < 0.001), T3 by 2.9 ± 3.5 kg (ES:0.65, large, *p* = 0.023), and T0 by 7.1 ± 4.7 kg (ES:1.16, large, *p* < 0.001). FM (kg) also decreased at T6 compared to T0 by 3.8 ± 3.1 kg (ES:0.75, large, *p* < 0.001) and at T3 compared to T0 by 3.9 ± 2.4 kg (ES:0.46, medium, *p* < 0.001). FFM did not change significantly.

V̇O_2_max (L·min^−1^) increased by the time, in fact, at T12, it was higher compared to T3 by 0.5 ± 0.27 L·min^−1^ (ES:1.13, large, *p* < 0.001) and at T0 by 0.9 ± 0.40 L·min^−1^ (ES:2.22, large, *p* < 0.001). At T30, it was higher compared to T0 by 0.2 ± 1.50 L·min^−1^ (ES:1.04, large, *p* < 0.001). At T6, it was higher compared to T3 by 0.3 ± 0.30 L·min^−1^ (ES:0.69, large, *p* = 0.022) and T0 by 0.7 ± 0.34 L·min^−1^ (ES:1.75, large, *p* < 0.001). At T3, it was also higher than T0 by 0.5 ± 0.36 L·min^−1^ (ES:1.06, large, *p* < 0.001) (Table 1).

Friedman test analysis for IPAQ_VIG (MET-min·week⁻^1^) showed significant effects for IPAQ_VIG (χ^2^ = 31.98, *p* = 0.003, Kendall’s W = 1.142). Post hoc comparisons revealed IPAQ_VIG (MET-min·week⁻^1^) was higher at T12 compared to T3 by 57 ± 39 MET-min·week⁻^1^ (*p* < 0.001, ES = 0.07, small), and lower than at T6 by 128 ± 32 MET-min·week⁻^1^ (*p* < 0.001, ES = 0.05, small). SF12_PI scores at T12 were higher compared to T3 and T6 by 2.6 ± 0.3 pt (*p* < 0.001, ES = 0.41, medium) (Table 2).

Energy intake analysis across all time points revealed a significant effect (*p* < 0.001); however, post hoc analysis did not reveal significant differences between individual time points. Still, significant reductions were observed at T3, T6, and T12 compared to T0 by 1254 ± 1260 kcal·day⁻^1^ (*p* < 0.001, ES = 0.53, large), 994 ± 1188 kcal·day⁻^1^ (*p* = 0.044, ES = 0.40, medium), and 1204 ± 1131 kcal·day⁻^1^ (*p* = 0.002, ES = 0.63, large), respectively. At T30, intake was higher than T3 by 1259 ± 291 kcal·day⁻^1^ (*p* = 0.028) (Table 2).

### 3.2. Characteristics of the 15 Participants That Were Included in All Time Points of This Study

The analysis for the 15 participants present at all time points showed significant effects for BM (F = 3.869, *p* = 0.042, and η^2^ = 0.075). Post hoc comparisons revealed lower BM at T12 compared to T0 by 8.2 ± 7.1 kg (*p* = 0.031, ES = 0.83, large), T3 by 5.3 ± 4.4 kg (*p* = 0.026, ES = 0.55, large), and T6 by 3.3 ± 2.7 kg (*p* = 0.027, ES = 0.32, medium) (Table 3). BMI followed a similar trend with significantly lower values at T12 vs. T0 by 2.80 ± 1.60 kg·m⁻^2^ (*p* = 0.019, ES = 1.33, large), T3 by 1.88 ± 2.30 kg·m⁻^2^ (*p* = 0.012, ES = 0.97, large), and T6 by 1.06 ± 2.50 kg·m⁻^2^ (*p* = 0.030, ES = 0.46, medium).

FM (kg) showed a significant time effect (F = 4.295, *p* = 0.044, η^2^ = 0.131), was significantly reduced at T3 by 4.5 ± 2.9 kg (*p* = 0.044, ES = 0.86, large), T6 by 4.5 ± 3.3 kg (*p* = 0.011, ES = 0.76, large), and T12 compared to T0 by 8.0 ± 5.1 kg (*p* = 0.004, ES = 1.27, large). FM was also lower at T12 compared to T3 by 3.5 ± 3.2 kg (*p* = 0.048, ES = 0.56, large) and to T6 by 3.5 ± 2.6 kg (*p* = 0.012, ES = 0.52, large) (Table 3).

FM (%) showed a significant time effect (F = 4.336, *p* = 0.044, and η^2^ = 0.154). FM (%) was lower at T3, T6, and T12 compared to T0 by 3.0 ± 1.9% (*p* = 0.004, ES = 0.96, large), 2.6 ± 2.3% (*p* = 0.045, ES = 0.72, large), and 5.3 ± 3.6% (*p* = 0.006, ES = 1.34, large), respectively. Additionally, FM (%) was lower at T12 than at T6 by 2.7 ± 1.9% (*p* = 0.008, ES = 0.61, large) (Table 3).

V̇O_2_max (L·min⁻^1^) showed a significant time effect (F = 15.30, *p* = 0.001, and η^2^ = 0.319). It was significantly increased at T3 by 0.61 ± 0.23 L·min⁻^1^ (*p* < 0.001, ES = 1.50, large), T6 by 0.85 ± 0.41 L·min⁻^1^ (*p* < 0.001, ES = 1.82, large), and T12 by 0.91 ± 0.49 L·min⁻^1^ (*p* = 0.001, ES = 1.80, large) compared to T0. At T30, V̇O_2_max was still elevated compared to T0 by 0.60 ± 0.58 L·min⁻^1^ (*p* = 0.043, ES = 1.13, large).

V̇O_2_max normalized for FFM (ml·kg⁻^1^ FFM·min⁻^1^) showed a significant time effect (F = 31.10, *p* < 0.001, and η^2^ = 0.461). It increased significantly at T3 by 8.2 ± 4.6 (*p* = 0.001, ES = 1.45, large), T6 by 14.1 ± 5.4 (*p* < 0.001, ES = 2.33, large), and T12 by 14.5 ± 6.9 (*p* < 0.001, ES = 2.24, large) compared to T0. At T30, it was still higher than T0 by 6.6 ± 6.5 (*p* < 0.001, ES = 0.99, large), but lower than T6 by 7.5 ± 5.4 (*p* = 0.001, ES = 1.20, large) (Table 3).

Friedman test analysis for IPAQ_TOT (MET-min·week⁻^1^) showed significant effects for IPAQ_TOT (χ^2^ = 14.41, *p* = 0.006, and Kendall’s W = 0.240). IPAQ_TOT increased significantly at T12 vs. T0 by 858 ± 2269 MET-min·week⁻^1^ (*p* = 0.015, ES = 0.54, medium), T3 by 60 ± 97 (*p* = 0.043, ES = 0.05, small), and T6 by 256 ± 135 (*p* = 0.001, ES = 0.34, small), and remained higher at T30 vs. T0 by 960 ± 2086 (*p* = 0.024, ES = 0.68, large) (Table 4).

Friedman test analysis for IPAQ_VIG (MET-min·week⁻^1^) showed significant effects for IPAQ_VIG (χ^2^ = 10.21, *p* = 0.037, and Kendall’s W = 0.170). IPAQ_VIG was significantly higher at T30 vs. T0 by 376 ± 1440 (*p* = 0.043, ES = 0.60, large) and T6 by 315 ± 2040 (*p* = 0.038, ES = 0.55, large).

Friedman test analysis for IPAQ_MOD (MET-min·week⁻^1^) showed significant effects for IPAQ_MOD (χ^2^ = 20.50, *p* < 0.001, and Kendall’s W = 0.342). IPAQ_MOD was significantly increased at T12 vs. T0 by 192 ± 400 (*p* < 0.001, ES = 0.70, large) and T30 by 52 ± 360 (*p* = 0.002, ES = 0.34, medium).

IPAQ_WALK did not change significantly across time (*p* = 0.101).

SF12_PI (pt) showed a significantly time effect (F = 3.775, *p* = 0.037, and η^2^ = 0.123). At T12, SF12_PI was higher than at T0 by 5.7 ± 6.5 pt (*p* = 0.045, ES = 1.04, large), T3 by 2.6 ± 0.2 pt (*p* < 0.001, ES = 0.82, large), and T6 by 4.6 ± 0.4 pt (*p* < 0.001, ES = 0.83, large) (Table 4).

SF12_MI (pt) showed a significant time effect (F = 5.559, *p* = 0.013, and η^2^ = 0.093). SF12_MI at T12 was significantly higher than T3 by 2.3 ± 0.4 pt (*p* < 0.001, ES = 0.31, medium) and T6 by 6.6 ± 0.2 pt (*p* < 0.001, ES = 1.00, large), and also higher at T30 compared to T0 by 7.2 ± 7.7 pt (*p* = 0.028, ES = 0.73, large).

Analysis of energy intake (kcal·day^−1^) across all time points showed statistical significance (*p* = 0.047); however, post hoc analysis revealed no significant difference between the individual time points.

### 3.3. Univariate and Multivariate Analysis of 15 Participants That Were Included in All Time Points of This Study

For the 15 subjects who participated in all time points of the study, we performed univariate and multivariate analyses using BMI, FM (%), and V̇O_2_max as dependent variables, while age, IPAQ_TOT score, energy intake, SF12_MI, and SF12_PI scores were defined as independent variables (Table 5).

The univariate analysis of BMI showed that it correlated negatively with age (β = −0.21, *p* < 0.001), the IPAQ_TOT score (β = −0.42, *p* = 0.041), and the results of the questionnaire (β = −0.09, *p* = 0.022) for SF12_MI and SF12_PI (β = −0.16, *p* = 0.002) (Table 5). The multivariate analysis of BMI showed that age (β = −0.19, *p* = 0.005) and the SF12_PI score (β = −0.12, *p* = 0.032) have the greatest influence on the BMI variations considering all the time points (Table 5).

The univariate analysis of FM (%) showed that it correlated negatively with the IPAQ TOT score (β = −1.34, *p* = 0.008) and the result of the SF12_MI questionnaire (β = −0.26, *p* = 0.004) (Table 6). The multivariate analysis of FM (%) showed that IPAQ_TOT score (β = −1.36, *p* = 0.001), energy intake (kcal·day^−1^) (β = −2.09, *p* < 0.001), and SF 12_MI (β= −0.24, *p* = 0.010) have the greatest influence on the variations in FM (%) considering all the time points (Table 6).

The univariate analysis of V̇O_2_max (L·min^−1^) showed that it correlated negatively with age (β = −0.04, *p* = 0.017) and positively with IPAQ_TOT score (β = 0.16, *p* = 0.003), SF 12_MI score (β = 0.04, *p* < 0.001), and SF 12_PI score (β = 0.04, *p* = 0.004) questionnaires (Table 7). The multivariate analysis of V̇O_2_max (L·min^−1^) showed that age (β = −0.08, *p* < 0.001), IPAQ_TOT score (β = 0.15, *p* < 0.001), and SF 12_MI (β = 0.03, *p* = 0.001) had the largest influence on the variation in V̇O_2_max when all time points were considered (L·min^−1^) (Table 7).

## 4. Discussion

The present study was aimed to evaluate body composition, cardiorespiratory fitness, physical activity levels, and physical and mental health 18 months after the end of a 12-month aerobic training program in a group of male adults with obesity. The study shows that a 12-month training program in male adults with obesity resulted in relevant adherence to training (61.8%) considering the initial number of participants at T0. Indeed, 21/34 participants completed the training period with beneficial effects on BM, FM, FFM, and V̇O_2_max. This was followed by a decrease in adherence (42.8%, 15/34 participants) at T30. Six of twenty-one participants did not take part in the follow-up examination because they did not comply with our request for follow-up examinations. However, the participants who completed this last part of the study maintained higher V̇O_2_max values than T0. In addition, participants who completed all time points showed significant reduction in BM, BMI, and FM at T12, while FFM was maintained, and V̇O_2_max increased; and at T30, V̇O_2_max maintained higher values than T0.

Adherence observed in this study at T12 (61.8%) and at T30 (42.8%) was in line with previous studies that showed follow-up participation rates that ranged between 30% and 62% [21,39]. In addition, all participants reduced BM and FM from T0 to T12, while maintaining FFM. Previous findings associate these adaptations with a decrease in mortality rates for cardiovascular and metabolic diseases [40]. However, the reduction in BM and FM throughout the training period was reversed during the follow-up period (T30). This is consistent with Zhang et al. [41], who showed that BM returned to baseline values one year after the end of a weight loss program in a group of adults with obesity enrolled in both moderate and vigorous exercise groups [41]. Conversely, we observed improvements in V̇O_2_max during the training period (T12) that remained higher in the follow-up period (T30) compared to T0, which is consistent with an improvement in health status. As previously observed by Wills et al. [42], participants who had higher CRF were associated with a lower mortality risk and longer life expectancy [6]. To our knowledge, our study was the first to show that combining HIIT and MICT with a polarized approach was a viable alternative for maintaining the CRF improvements in adults with obesity, compared to HIIT and MICT alone during the follow-up period. This suggests that physical activity with a combination of high volume at moderate intensity and low volume at vigorous intensity, maintained during the follow-up period, was useful to maintain or increase V̇O_2_max over a long period of time [43].

When considering the participants who completed all testing sessions, 12 months of training resulted in a loss of 8% of BM, 21% FM (kg) and maintenance of FFM, which was considered successful in people with obesity [44]. Similarly, the study of Quist et al. [45] showed that long-term active commuting of three different types of exercise (e.g., 50% V̇O_2_ peak for moderate intensity, and 70% of V̇O_2_ peak for vigorous intensity and active bicycle commuting to and from work), five times per week, did not result in significant BM changes, but did result in reduced FM [45]. Hemmingson’s study pointed out that changing participants’ lifestyle for 6 months did not lead to BM changes in people with overweight or obesity [46]. This suggests that an individualized training program leads to greater improvements than lifestyle changes alone. Furthermore, our study shows that V̇O_2_max at T12 was 29.6% higher in absolute terms and 30.0% higher normalized to kg of FFM than at T0, which is consistent with previous studies. Indeed, Schleh [47] showed that 12 weeks of HIIT or MICT training in adults with obesity and metabolic syndrome improved anthropometric characteristics (weight loss and FM), and V̇O_2_max, especially in those who performed HIIT [47]. In a 16-week study in which people trained three times a week, Poon et al. [48] showed that both MICT and HIIT training led to an improvement in aerobic fitness, but that the combined training shows better training adherence [48].

In addition, in the present study, V̇O_2_max was maintained higher at T30 than at T0, which is consistent with the study of Quist et al. [21], which showed that 6 months of cycling (i.e., active commuting) and vigorous exercise training (i.e., 70% of V̇O_2_ peak) was associated with a long-term maintenance of V̇O_2_peak for approximately 15 months after the end of the training program [21]. A 10% improvement in V̇O_2_peak [49] and a 5% reduction in FM [44] have been found to represent significant improvements for CRF and body composition in people with obesity.

In our study, V̇O_2_max at T30 was higher (+ 19.5%) than at T0, but FM returned similar to baseline and FFM remained stable. The participants in the follow-up study stated in an interview that they continued to exercise after the end of the study, although not as frequently as before and not as often per week. Despite the decrease in exercise sessions, V̇O_2_max remained higher than T0 and FFM did not decrease, leading us to confirm the central role of physical activity in the health of people with obesity [11].

Previous studies suggest that individuals with lower fitness levels, such as many people with obesity, tend to have greater variability in maintaining V̇O_2_max values after the end the training period [50]. However, the general trend was a decrease in aerobic capacity, which may further compromise metabolic and cardiovascular health [51]. In our study, V̇O_2_max remained higher at T30 than T0, suggesting that combined training may be useful to maintain a good CRF that improves quality of life, as previously shown [52].

In our study, the participants who completed all experimental time points were those who exercised more at low intensity (70–90% of weekly training) and completed 2 to 3 low-intensity exercise sessions per week during the follow-up period. This is consistent with studies in endurance athletes that have shown greater long-term effectiveness of low-intensity, high-volume exercise in maintaining cardiovascular fitness and metabolic health [53,54]. From a physiological perspective, it appears that exercise volume rather than exercise intensity could be the important factor in improving mitochondrial content (i.e., a peripheral factor of V̇O_2_max) [55]. In contrast, exercise intensity is an important factor in improving mitochondrial respiration [4,16]. Although few studies investigated the central or peripheral factors in improving aerobic capacity in patients with obesity, it is primarily the role of mitochondria in fat and carbohydrate oxidation during resting and exercise conditions, as obesity led to metabolic inflexibility [56,57]. Thus, in support of the above, increased weekly levels of physical activity, as observed in IPAQ TOT and MOD scores, may have contributed to the maintenance or increase in V̇O_2_max at T30. This was confirmed by the significant relationship between V̇O_2_max and the IPAQ_TOT score considering all time points in the univariate analysis. This confirms the primary role of aerobic exercise in improving cardiorespiratory fitness in people with obesity [58].

Obesity has been associated with reduced quality of life, which has negative consequences for physical and mental health [59]. In our study, we observed that the physical and mental index scores of the SF_12 questionnaire at T30 were significantly higher than the baseline values. The data from the linear mixed model with univariate and multivariate analysis also suggest that the improvements in physical and mental health assessed by the SF_12 were significantly related to the reduction in FM (%) and the increase in V̇O_2_max. This may suggest that improving physical capacities and body composition enhances mental and physical well-being. This is consistent with a recent review suggesting that the improvements in CRF observed in individuals with obesity increase tolerance for daily activities, improve quality of life, and contribute to a reduction in depressive symptoms [60].

### 4.1. Limitations

The present study has some limitations that must be taken into account when interpreting the results. First, the lack of a control group limits the possibility of establishing a causal relationship between the specific training interventions and the observed effects. In addition, it might be interesting to analyze the effects of different training protocols for periods longer than 6 months.

### 4.2. Practical Applications

It is recommended that people with obesity follow a structured physical activity program, as this leads to an improvement in physical abilities and physical and mental well-being. A personalized and monitored approach can lead to greater adherence to treatment and more effective long-term outcomes.

## 5. Conclusions

Twelve months of an aerobic training program (i.e., POL or HIIT) in male adults with obesity resulted in relevant adherence (61.8%) with beneficial effects on BM, FM, FFM, and V̇O_2_max. Assessments at 30 months since the beginning of the study showed a reduction in adherence (42.8%) and a preservation of higher V̇O_2_max compared to T0. Maintaining physical activity during follow up (T12 to T30) was conceivably crucial to maintain the V̇O_2_max gains and the improved perception of quality of life.

## Figures and Tables

**Table 1:** Anthropometric characteristics and physical capacities measured during all the time points in all participants.

	T0 (n: 34)	T3 (n: 34)	T6 (n: 28)	T12 (n: 21)	T30 (n: 15)	*p* Value
Age (y)	39.5 ± 7.0	39.8 ± 7.0	41.3 ± 6.3	41.7 ± 6.3	43.1 ± 6.9	0.386
Body mass (kg)	105.3 ± 13.2	102.2 ± 13.4 *	98.7 ± 10.0 *^†^	96.1 ± 10.8 *^†£^	99.0 ± 11.4	0.001
BMI (kg m^−2^)	33.9 ± 4.2	32.9 ± 4.2 *	31.6 ± 2.6 *^†^	30.7 ± 2.6 *^†£^	31.3 ± 3.7	0.001
Fat-free mass (kg)	64.2 ± 7.1	65.0 ± 6.9	63.1 ± 5.3^†^	63.9 ± 5.9	65.7 ± 4.6	0.009
Fat mass (kg)	41.1 ± 8.5	37.2 ± 8.6 *	35.5 ± 6.2 *	32.2 ± 6.9 *^†£^	33.3 ± 9.8	0.001
Fat mass (%)	38.8 ± 4.1	36.3 ± 4.2 *	35.8 ± 3.5 *	33.0 ± 4.6 *^†£^	33.1 ± 6.6	0.001
V̇O_2_max (L·min^−1^)	3.02 ± 0.44	3.49 ± 0.46 *	3.79 ± 0.44 *^†^	4.01 ± 0.45 *^†^	3.59 ± 0.64 *^&^	0.001
V̇O_2_max (mL·kg^−1^ FFM ·min^−1^)	47.2 ± 5.8	53.9 ± 6.2 *	60.1 ± 5.8 *^†^	63.1 ± 6.7 *^†^	54.5 ± 7.0 *^&^	0.001

All values are presented as mean ± standard deviation. BMI: body mass index, V̇O_2_peak: peak oxygen uptake, V̇O_2_peak FFM-1: peak oxygen uptake normalized by fat-free mass; * different vs. T0 *p* < 0.05; ^†^ different vs. T3 *p* < 0.05; ^£^ different vs. T6 *p* < 0.05; and ^&^ different vs. T12 *p* < 0.05.

**Table 2:** Physical activity habits and energy intake measured during all the time points in all participants.

	T0 (n: 34)	T3 (n: 34)	T6 (n: 28)	T12 (n: 21)	T30 (n: 15)	*p* Value
IPAQ_TOT (MET-min·week^−1^)	1413 (6812)	1815 (7029)	1662 (4704)	1906 (7380)	1857 (8748)	0.066
IPAQ_VIG (MET-min·week^−1^)	1020 (2160)	1080 (3552)	1280 (2592)	1134 (2722) ^†£^	1440 (4560)	0.002
IPAQ_MOD (MET-min·week^−1^)	440 (920)	380 (2880)	400 (2780) ^†^	420 (2919) ^†£^	720 (1800)	0.066
IPAQ_WALK (MET-min·week^−1^)	553 (5544)	594 (3366)	495 (3881) ^†^	624 (3534) ^†£^	396 (5412)	0.326
SF12_PI (pt)	49.8 ± 7.5	51.3 ± 6.2	52.6 ± 4.0	54.0 ± 6.5 ^†£^	50.1 ± 7.8	0.038
SF12_MI (pt)	43.7 ± 10.0	45.0 ± 10.7	46.7 ± 9.7	47.2 ± 11.2	48.6 ± 11.0	0.191
Energy intake (kJ·day^−1^)	8700 ± 2280	7546 ± 2100 *	7830 ± 2071 *^†^	7434 ± 1692 *	8805 ± 1809 ^†^	0.001

The values are presented as mean ± standard deviation or median and interquartile range in the case of data distributed in a non-normal way. IPAQ_TOT: International Physical Activity Questionnaire, IPAQ_VIG: vigorous activity, IPAQ_MOD: moderate-intensity activity, IPAQ_WALK: physical activity derived from walking, SF12_PI: Short-Form 12, questionnaire about health-related quality of life concerning physical index, and SF12_MI: Short-Form 12, questionnaire about health-related quality of life concerning mental index. * different vs. T0 *p* < 0.05; ^†^ different vs. T3 *p* < 0.05; ^£^ different vs. T6 *p* < 0.05.

**Table 3:** Anthropometric characteristics and physical capacities measured during all the time points in the participants (n: 15) who complete all time points.

	T0 (n: 15)	T3 (n: 15)	T6 (n: 15)	T12 (n: 15)	T30 (n: 15)	*p* Value
Age (y)	39.8 ± 8.1	40.1 ± 8.1 *	40.8 ± 8.1 *	41.2 ± 8.1 *^£^	42.8 ± 8.1 *^†£&^	0.001
Body mass (kg)	102.4 ± 8.8	99.5 ± 8.4	97.4 ± 9.7	94.1 ± 10.8 *^†£^	99.0 ± 11.4	0.042
BMI (kg m^−2^)	33.0 ± 1.6	32.1 ± 1.8	31.3 ± 2.3	30.2 ± 2.5 *^†£^	31.5 ± 3.7	0.023
Fat-free mass (kg)	63.6 ± 4.6	65.2 ± 4.0	62.9 ± 5.3 ^†^	63.4 ± 5.8	65.7 ± 4.6	0.054
Fat Mass (kg)	38.7 ± 5.4	34.2 ± 5.1 *	34.3 ± 6.3 *	30.7 ± 7.1 *^†£^	33.3 ± 9.8	0.044
Fat Mass (%)	37.7 ± 3.0	34.7 ± 3.3 *	35.1 ± 4.1 *	32.4 ± 4.7 *^£^	33.1 ± 6.6	0.044
V̇O_2_max (L·min^−1^)	3.07 ± 0.46	3.69 ± 0.35 *	3.93 ± 0.48 *	3.98 ± 0.55 *	3.67 ± 0.60 *	0.001
V̇O_2_max (mL·kg^−1^ FFM·min^−1^)	48.3 ± 6.6	56.6 ± 4.7 *	62.4 ± 5.5 *^†^	62.8 ± 6.4 *^†^	54.5 ± 7.0 *^£&^	0.001

All values are presented as mean ± standard deviation.; BMI: body mass index, V̇O_2_peak: peak oxygen uptake, and V̇O_2_peak FFM-1: peak oxygen uptake normalized by fat-free mass. * different vs. T0 *p* < 0.05; ^†^ different vs. T3 *p* < 0.05; ^£^ different vs. T6 *p* < 0.05; and ^&^ different vs. T12 *p* < 0.05.

**Table 4:** Physical activity habits and energy intake measured during all the time points in the participants who complete all time points.

	T0 (n: 15)	T3 (n: 15)	T6 (n: 15)	T12 (n: 15)	T30 (n: 15)	*p* Value
IPAQ_TOT (MET-min week^−1^)	396 (2888)	1194 (5100)	998 (5448)	1254 (5355) *^†£^	1356 (9144) *	0.006
IPAQ_VIG (MET-min week^−1^)	584 (2400)	720 (3840)	645 (2760)	378 (2016)	960 (4800) *^£^	0.037
IPAQ_MOD (MET-min week^−1^)	60 (720)	240 (2400)	198 (2195)	252 (2520) *	200 (2160) ^&^	0.001
IPAQ_WALK (MET-min week^−1^)	198 (1040)	396 (1980)	325 (1912)	416 (2079)	132 (5544)	0.101
SF12_PI (pt)	48.8 ± 7.0	51.9 ± 3.1	49.9 ± 2.8	54.5 ± 3.4 *^†£^	50.1 ± 7.8	0.037
SF12_MI (pt)	41.4 ± 8.9	46.3 ± 7.4	42.1 ± 5.3	48.7 ± 7.6 ^†£^	48.6 ± 11.0 *	0.013
Energy intake (kJ·day^−1^)	9012 ± 1619	8014 ± 1521	8845 ± 1356	8046 ± 1431	8805 ± 1809	0.047

The values are presented as mean ± standard deviation or median and interquartile range in the case of data distributed in a non-normal way. IPAQ_TOT: International Physical Activity Questionnaire, IPAQ_VIG: vigorous activity, IPAQ_MOD: moderate-intensity activity, IPAQWALK: physical activity derived from walking, SF12_PI: Short-Form 12, questionnaire about health-related quality of life concerning physical index, and SF12_MI: Short-Form 12, questionnaire about health-related quality of life concerning mental index. * different vs. T0 *p* < 0.05; ^†^ different vs. T3 *p* < 0.05; ^£^ different vs. T6 *p* < 0.05; and ^&^ different vs. T12 *p* < 0.05.

**Table 5:** Linear mixed model univariate and multivariate of the dependent variable BMI in comparison to the independent variables.

	Univariate Analysis			Multivariate Analysis		
BMI (kg m^−2^)	Beta Coefficient	95% CI	*p*-Value	Beta Coefficient	95% CI	*p*-Value
Age (y)	−0.21	−0.32, −0.10	**<0.001**	−0.19	−0.32, −0.05	**0.005**
IPAQ_TOT (MET-min·week^−1^)	−0.42	−0.83, −0.02	**0.041**	−0.23	−0.55, 0.09	0.157
Energy intake (kJ·day^−1^)	0.02	−0.47, 0.51	0.936			
SF 12 MI (pt)	−0.09	−0.16, −0.01	**0.022**	0.01	−0.07, 0.08	0.929
SF 12 PI (pt)	−0.16	−0.26, −0.06	**0.002**	−0.12	−0.23, −0.01	**0.032**

The values are presented as beta coefficient and 95%CI. Bold text indicates a significant relationship. BMI: body mass index, IPAQ_TOT: International Physical Activity Questionnaire, SF12_PI: Short-Form 12, questionnaire about health-related quality of life concerning physical index, and SF12_MI: Short-Form 12, questionnaire about health-related quality of life concerning mental index.

**Table 6:** Linear mixed model univariate and multivariate of the dependent variable FM (%) in comparison to the independent variables.

	Univariate Analysis			Multivariate Analysis		
FM (%)	Beta Coefficient	95% CI	*p*-Value	Beta Coefficient	95% CI	*p*-Value
Age (y)	−0.29	−0.66, 0.09	0.136			
IPAQ_TOT (MET-min·week^−1^)	−1.34	−2.33, −0.34	**0.008**	−1.36	−2.16, −0.56	**0.001**
Energy intake (kJ·day^−1^)	−1.01	−2.20, 0.17	0.094	−2.09	−3.05, −1.13	**<0.001**
SF 12 MI (pt)	−0.26	−0.43, −0.08	**0.004**	−0.24	−0.43, −0.06	**0.010**
SF 12 PI (pt)	−0.20	−0.46, 0.06	0.137			

The values are presented as beta coefficient and 95%CI. Bold text indicates significant relationship. FM: fat mass, V̇O_2_max: max oxygen uptake, IPAQ_TOT: International Physical Activity Questionnaire, SF12_PI: Short-Form 12, questionnaire about health-related quality of life concerning physical index, and SF12_MI: Short-Form 12, questionnaire about health-related quality of life concerning mental index.

**Table 7:** Linear mixed model univariate and multivariate of the dependent variable V̇O_2_max in comparison to the independent variables.

	Univariate Analysis			Multivariate Analysis		
V̇O_2_max (L min^−1^)	Beta Coefficient	95% CI	*p*-Value	Beta Coefficient	95% CI	*p*-Value
Age (y)	−0.04	−0.08, 0.01	**0.017**	−0.08	−0.11, −0.05	**<0.001**
IPAQ_TOT (MET-min·week^−1^)	0.16	0.05, 0.26	**0.003**	0.15	0.08, 0.22	**<0.001**
Energy intake (kJ·day^−1^)	−0.08	−0.20, 0.05	0.228			
SF 12 MI (pt)	0.04	0.02, 0.07	**<0.001**	0.03	0.01, 0.05	**0.001**
SF 12 PI (pt)	0.04	0.01, 0.07	**0.004**	0.02	−0.01, 0.05	0.097

The values are presented as beta coefficient and 95%CI. Bold text indicates significant relationship. V̇O_2_max: max oxygen uptake, IPAQ_TOT: International Physical Activity Questionnaire, SF12_PI: Short-Form 12, questionnaire about health-related quality of life concerning physical index, and SF12_MI: Short-Form 12, questionnaire about health-related quality of life concerning mental index.

## Data Availability

The corresponding author is available to share the primary data to those interested.

## Abbreviations

The following abbreviations are used in this manuscript:

BM	Body mass
BMI	Body mass index
V̇O_2_max	Max oxygen uptake
IPAQ_TOT	International Physical Activity Questionnaire
FM	Fat mass
FFM	Fat-free mass
POL	Polarized training
GET	Gas exchange threshold
RCP	Respiratory compensation point
HR	Heart rate
HIIT	High intensity interval training
COMB	Combined training
THR	Threshold training
GRAD	Graded exercise test
CRF	Cardiorespiratory fitness
